# Successful treatment of ulceration in hidradenitis suppurativa with topical bucladesine: A case report

**DOI:** 10.1002/ski2.321

**Published:** 2023-12-08

**Authors:** Ichiro Kurokawa, Kanako Kita, Takashi Hashimoto

**Affiliations:** ^1^ Department of Dermatology Meiwa Hospital Nishinomiya Hyogo Japan; ^2^ Department of Dermatology Osaka Metropolitan University, Graduate School of Medicine Osaka Japan

## Abstract

We report a case of hidradenitis suppurativa (HS) with skin ulceration in a 19‐year‐old man. He was successfully treated with topical bucladesine ointment treatment, resulting in a hypertrophic scar 2 months after the treatment. Bucladesine can be an alternative treatment option for ulceration in HS.

## Graphical abstract

We report a case of successful treatment of ulceration in hidradenitis suppurativa (HS) with topical bucladesine. The ulcer re‐epithelialised over 2 months with topical bucladesine ointment application, resulting in a hypertrophic scar. We propose bucladesine can be an alternative treatment option for ulceration in HS.

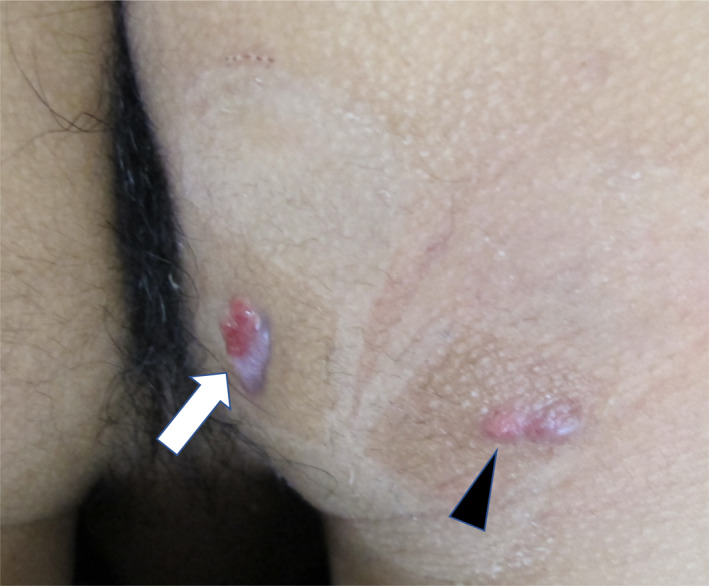

## 1 INTRODUCTION

Hidradenitis suppurativa (HS) is a refractory follicular occlusion disease characterised by painful deep‐seated recurrent subcutaneous nodules, fistulas, abscesses, and sinus tracts.^1^ HS often manifests as ulceration; however, its pathogenesis remains unclear. The treatment of ulceration in HS has not been well studied. Topical treatments, such as antimicrobials and antiseptic agents, have been used for HS but appear inefficient. The aetiology of HS remains unclear, but autoinflammation and abnormal keratinisation is involved in the pathogenesis of HS.^2^

## 2 CASE REPORT

**FIGURE 1:**
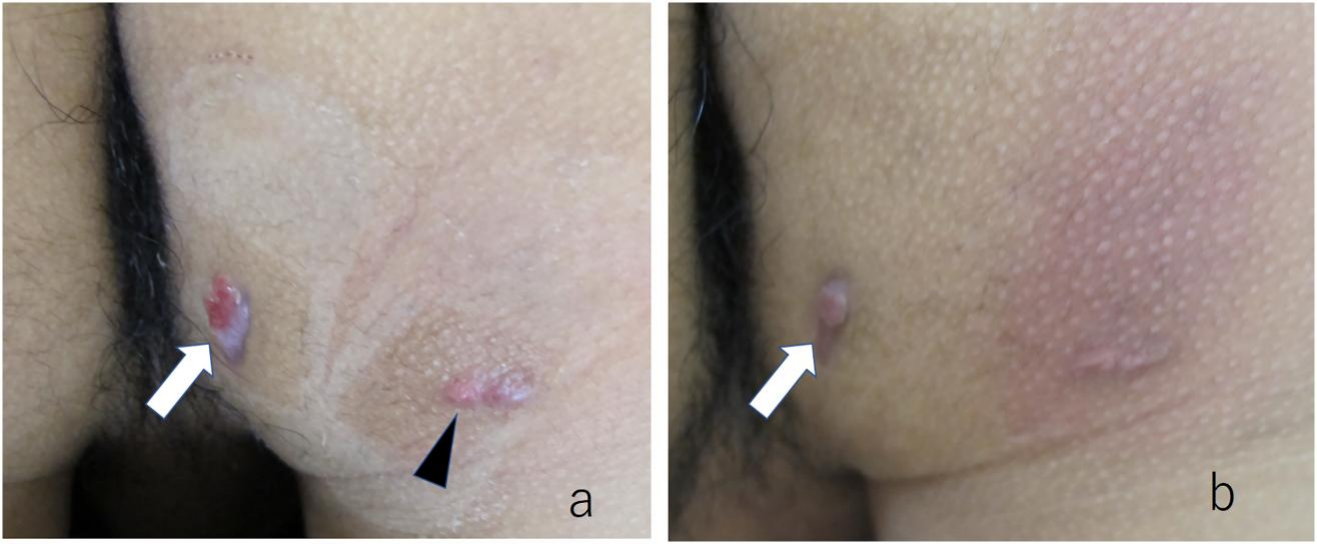
(a) Ulceration on the right glutaeal fold (white arrow) before treatment and hypertrophic scar (arrowhead) were observed; (b) After 2 months of topical bucladesine treatment, the ulceration was re‐epithelialised, resulting in formation of the hypertrophic scar (white arrow).

A 19‐year‐old male presented with a subcutaneous fistula on his buttock for 4 years. He had an episodic history of recurrent painful inflammatory subcutaneous nodules on his buttocks for 6 months. He had no lesions in axilla. The patient was treated with oral minocycline 100 mg/d and topical fusidic ointments. Although the subcutaneous draining fistula and sinus tract improved, the non‐draining fistula and the ulceration persisted (Figure 1a). The severity of this case was Hurley II. The score of International HS Severity Score System (IHS4) was 5. The ulcer re‐epithelialised over 2 months with topical bucladesine ointment treatment, resulting in a hypertrophic scar (Figure 1b). The patient did not experience recurrence 2 years after the treatment. Informed consent was obtained from the patient for reproduction of the clinical findings for publication.

## 3 DISCUSSION

Topical bucladesine has been used to treat decubitus and post‐burn ulcers. Bucladesine, which is metabolised into cyclic adenosine monophosphate in wound tissue, dilates the peripheral blood vessels, improves the blood supply, and promotes the proliferation of endothelial cells (angiogenesis) and the proliferation and migration of keratinocytes and fibroblasts.^3^ This results in granulation tissue formation, reepithelialisation, and scar formation. Here we report a case of HS with ulceration over the buttocks that was successfully treated with topical bucladesine ointment.

Topical bucladesine ointment effectively resolved ulceration in HS, resulting in the formation of a hypertrophic scar. Secondary intention healing has been reported.^4^ However, healing takes a long time. We speculate that bucladesine promotes secondary healing to form hypertrophic scars. Although the mechanism of bucladesine is unclear, we believe that it produces interleukin‐6 and transforming growth factor‐β by keratinocytes and fibroblast,^3^ resulting in re‐epithelialisation and hypertrophic scarring as secondary intention healing.^4^

Complications of squamous cell carcinoma with long‐standing HS lesions have been reported, especially in the male buttock.^5^ Therefore, we chose bucladesine instead of basic fibroblast growth factor because bucladesine is not carcinogenic. Nevertheless, long‐term follow‐up is necessary to identify the occurrence of squamous cell carcinoma.

Our findings suggest that bucladesine can be an alternative treatment option for ulceration in HS. Further case studies for more HS cases are warranted to validate the findings in this report and to clarify the effectiveness of bucladesine for ulceration of HS.

## CONFLICT OF INTEREST STATEMENT

None to declare.

## AUTHOR CONTRIBUTIONS

**Ichiro Kurokawa**: Conceptualisation (equal); Data curation (equal); Formal analysis (equal); Funding acquisition (equal); Investigation (equal); Methodology (equal); Project administration (equal); Resources (equal); Software (equal); Supervision (equal); Validation (equal); Visualisation (equal); Writing – original draft (equal); Writing – review & editing (equal). **Kanako Kita**: Conceptualisation (equal); Data curation (equal); Formal analysis (equal). **Takashi Hashimoto**: Conceptualisation (equal); Data curation (equal); Formal analysis (equal); Supervision (equal); Writing – original draft (equal); Writing – review & editing (equal).

## ETHICS STATEMENT

Informed consent was approved by IRB in Meiwa Hospital.

## Data Availability

The data that support the findings of this study are available on request from the corresponding author. The data are not publicly available due to privacy or ethical restrictions.
